# *CDH13* promoter region is specifically methylated in poorly differentiated colorectal cancer

**DOI:** 10.1038/sj.bjc.6601647

**Published:** 2004-03-02

**Authors:** K Hibi, H Nakayama, Y Kodera, K Ito, S Akiyama, A Nakao

**Affiliations:** 1 Gastroenterological Surgery, Nagoya University Graduate School of Medicine, 65 Tsurumai-cho, Showa-ku, Nagoya 466-8560, Japan

**Keywords:** *CDH13*, colorectal cancer, methylation-specific PCR

## Abstract

It has recently become clear that *CDH13* (H-cadherin, T-cadherin) expression is frequently silenced by aberrant methylation in colorectal cancers and adenomas. In this study, we investigated the methylation status of *CDH13* gene and detected aberrant promoter methylation in 27 of 84 (32%) colorectal cancers. We then correlated the results with the clinicopathological features of affected patients. We found a significant difference in histology (*P*=0.0053) when we compared the *CDH13* methylation of poorly differentiated colorectal cancers to that of differentiated ones. This result suggested that poorly differentiated colorectal cancers specifically exhibited *CDH13* methylation, and since *CDH13* might be responsible for selective cell recognition and adhesion, inactivation of *CDH13* could lead to the formation of scattered carcinoma cells in these cancers.

There is now good evidence that a series of genetic alterations in both dominant oncogenes and tumour suppressor genes are involved in the pathogenesis of human colorectal cancer. Activation of oncogenes such as the *ras* gene, and inactivation of tumour suppressor genes such as the *APC* and *p53* genes have been identified in colorectal cancer (Bos *et al*, 1987; Baker *et al*, 1990; Nishisho *et al*, 1991). In addition, we found that several other genes are related to the pathogenesis of colorectal cancer (Hibi *et al*, 1996; Hibi *et al*, 1997; Hibi *et al*, 2002; Yamazaki *et al*, 2002). An investigation of genetic changes is important to clarify the tumorigenic pathway of colorectal cancer (Vogelstein *et al*, 1988).

Several tumour suppressor genes contain CpG islands in their promoters, prompting many studies that investigate the role of methylation in silencing these genes. Many tumour suppressor genes show evidence of methylation silencing, providing a new potential pathway for the deactivation of tumour suppressor genes (Herman *et al*, 1996). It has recently become clear that *CDH13* (H-cadherin, T-cadherin) expression is frequently silenced by aberrant methylation in colorectal cancers and adenomas (Toyooka *et al*, 2002). *CDH13* encodes a protein belonging to the cadherin family of cell surface glycoproteins responsible for selective cell recognition and adhesion (Takeichi, 1991). Ubiquitous methylation of *CDH13* in colorectal cancers and adenomas indicated that such methylation occurs at an early stage in the multistage process of oncogenesis. However, we do not yet know what roles *CDH13* methylation play in colorectal cancers.

In this study, we investigated the methylation status of *CDH13* in 84 colorectal cancers that were examined pathologically. We then correlated the results with the clinicopathological features of affected patients.

## MATERIALS AND METHODS

### Sample collection and DNA preparation

A total of 84 primary tumours and corresponding colorectal epithelial tissues were collected at the Nagoya University School of Medicine from Japanese colorectal cancer patients who had been diagnosed histologically. These samples were obtained during surgery. All tissues were quickly frozen in liquid nitrogen and stored at −80°C until analysis. Tumour and normal tissue samples were digested overnight by proteinase K, and DNA was prepared by extraction with phenol. Oral or written informed consent, as indicated by the institutional review board, was obtained from all patients. There was no family history about cancers in poorly differentiated colorectal cancer patients. Tumour sites of six poorly differentiated colorectal cancers were rectum (three patients), sigmoid colon (one patient), and cecum (two patients).

### Bisulphite modification and methylation-specific PCR (MSP)

DNA from tumour and normal tissue specimens was subjected to bisulphite treatment as described previously (Hibi *et al*, 2001). The modified DNA was used as a template for MSP. Primer sequences of *CDH13* for amplification were described previously (Sato *et al*, 1998). The primers for the unmethylated reaction were: CDH13UMS (sense), 5′-TTGTGGGGTTGTTTTTTGT, and CDH13UMAS (antisense), 5′-AACTTTTCATTCATACACACA, which amplify a 242 bp product. The primers for the methylated reaction were: CDH13MS (sense), 5′- TCGCGGGGTTCGTTTTTCGC, and CDH13MAS (antisense), 5′-GACGTTTTCATTCATACACGCG, which amplify a 243 bp product. The PCR amplification of modified DNA samples consisted of one cycle of 95°C for 5 min; 33 cycles of 95°C for 30 s, 60°C for 1 min, and 72°C for 1 min for the unmethylated reaction, or 29 cycles of 95°C for 30 s, 70°C for 1 min, and 72°C for 1 min for the methylated reaction; one cycle of 72°C for 5 min. DNAs from TE1 (oesophageal squamous cell cancer cell line) and SW1417 (colon cancer cell line) were used as positive controls for *CDH13* amplification of unmethylated and methylated alleles, respectively. The methylation status of SW1417 cells has been examined previously (Toyooka *et al*, 2002). Control reactions without DNA were performed for each set of PCR. A measure of 10 *μ*l of each PCR product was directly loaded onto nondenaturing 6% polyacrylamide gels, stained with ethidium bromide, and visualised under UV illumination. Each MSP was repeated at least three times. We considered that the presence of a visible PCR product in lane U or M indicated the presence of unmethylated or methylated genes, respectively.

### Reverse transcription (RT)–PCR

First strand cDNA was generated from RNA as described previously (Hibi *et al*, 1991). The PCR amplification consisted of 30 cycles (95°C for 30 s, 55°C for 1 min, and 72°C for 1 min) after the initial denaturation step (95°C for 2 min). The primers used were CDH13-S (sense), 5′-TTCAGCAGAAAGTGTTCCATAT, and CDH13-AS (antisense), 5′-GTGCATGGACGAACAGAGT. Primer sequences were described previously (Sato *et al*, 1998). The predicted size of the PCR product was 208 bp. The housekeeping gene, *β*-actin, was used as an internal control to confirm the success of the RT reaction.

### Statistical analysis

The *χ*^2^ test and Student^'^s *t*-test were used to examine the association between *CDH13* promoter methylation and clinicopathological features.

## RESULTS

**Figure 1:**
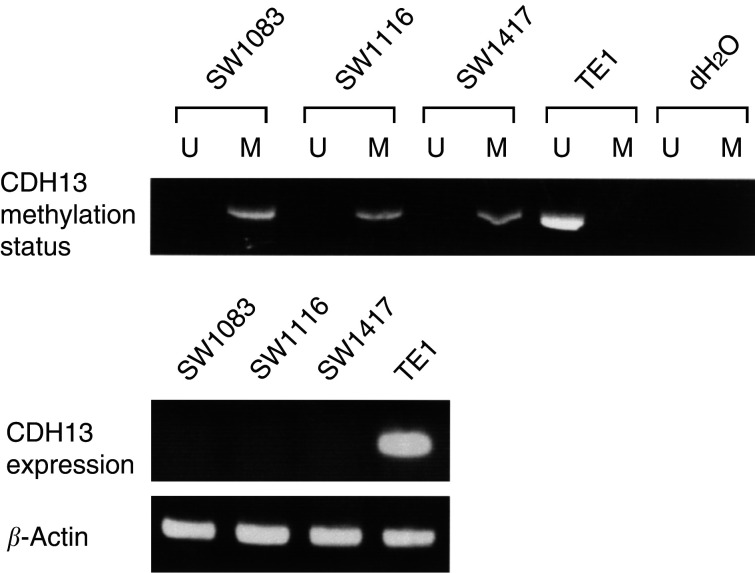
Representative MSP of *CDH13* promoter in colorectal cancer cell lines (SW1083, SW1116, and SW1417) and oesophageal cancer cell line (TE1). The presence of a visible PCR product in lane U indicates the presence of unmethylated genes; the presence of PCR product in lane M indicates the presence of methylated genes. All three colorectal cancer cell lines that demonstrated only methylation of the *CDH13* promoter lacked *CDH13* gene expression as determined by RT–PCR, while *CDH13* was expressed in TE1 with unmethylation of the *CDH13* promoter.

We first examined the methylation status of *CDH13* in colorectal cancer cell lines (SW1083, SW1116, and SW1417) and an oesophageal squamous cell cancer cell line (TE1) using MSP. DNA from all colorectal cancer cell lines exhibited abnormal promoter methylation of *CDH13* gene (Figure 1

). To confirm the status of *CDH13* gene according to the methylation pattern, we next examined *CDH13* expression in these cell lines using RT–PCR. All colorectal cancer cell lines that demonstrated methylation of the *CDH13* promoter lacked *CDH13* gene expression, while *CDH13* was expressed in the oesophageal cancer cell line with unmethylation of the *CDH13*.

**Figure 2:**
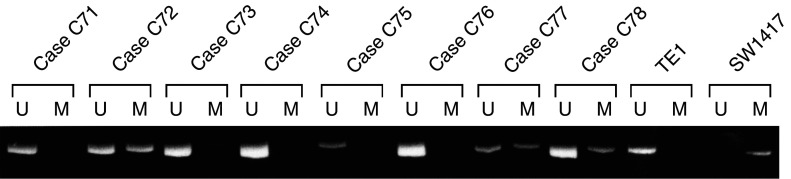
Representative MSP of *CDH13* promoter in colorectal cancer samples. *CDH13* promoter methylation was present in cases 72, 78, and 79. In each case, modified DNAs from TE1 and SW1417 were used as positive controls of *CDH13* for unmethylated and methylated alleles, respectively.

We next examined the methylation status of *CDH13* promoter in tumours using the MSP technique. Aberrant promoter methylation of the *CDH13* gene was detected in 27 of 84 (32%) colorectal cancers. This result indicated that *CDH13* aberrant methylation might play an important role in colorectal cancers, as described previously (Toyooka *et al*, 2002). According to the previous study, 49% of colorectal cancers that were collected from American colorectal cancer patients showed *CDH13* methylation. Colorectal cancers that we examined were collected from Japanese colorectal cancer patients. This might be the reason why there is a discrepancy of the ratio of *CDH13* methylation positivity between the previous and our studies. A representative MSP analysis of *CDH13* gene promoter methylation from tumours is shown in Figure 2

. As a control, we screened the DNA of 84 corresponding normal tissues for aberrant methylation, but found no methylation of *CDH13* in this group. Figure 2 showed no cases where methylation of colorectal cancers was complete. Therefore, it might be possible that the *CDH13* gene expression has not been inhibited completely in these cancers.

To determine the role of *CDH13* inactivation in colorectal cancer, we examined the correlation of methylation status with the clinicopathological features. There was no significant difference in the distribution of patients with positive or negative methylation of *CDH13* in terms of sex, maximal tumour size, the extent of tumour, lymph node metastasis, or Dukes' stage. However, we found a significant difference in histology (*P*=0.0053) when we compared the *CDH13* methylation of poorly differentiated colorectal cancers to that of other differentiated ones (Table 1

**Table 1:** Clinicopathological features and methylation status of *CDH13* promoter region in colorectal cancer patients

	**No. of cases**	***CDH13* methylation**		
**Clinicopathological variable features**		**+**	**−**	***P*-value**
Sex				
Male	45	18	27	0.098^a^
Female	39	9	30	

Maximal tumour size				
15–100 mm	84	50.9±21.3^b^	45.2±16.5	0.182^c^

Histology				
Poorly differentiated^d^	6	5	1	0.0053^a^
Other differentiated^e^	78	22	56	

Extent of tumour				
≦mt^f^	20	6	14	0.814^a^
mt<	64	21	43	

Lymph node metastasis				
+	31	6	25	0.055^a^
−	53	21	32	

Dukes' stage				
A, B	54	19	35	0.423^a^
C, D	30	8	22	

Total	84	27	57	

a *χ*^2^ test.

b Mean±s.d.

c Student^'^s *t*-test.

d Poorly differentiated or mucinous adenocarcinoma according to Japanese criteria.

e Well- or moderately differentiated adenocarcinoma according to Japanese criteria.

f Muscular tunic.

). These results suggest that poorly differentiated colorectal cancers specifically exhibited *CDH13* methylation.

## DISCUSSION

Colorectal cancer, one of the most aggressive cancers, occurs with a high incidence in most countries (Greenlee *et al*, 2000). To rid patients of this fatal cancer, tumours are resected and patients are then treated with chemotherapy and radiotherapy. To eliminate such cancers, it is also important to determine the genetic alterations as a new parameter for an estimation of colorectal cancer. Colorectal cancers are classified histopathologically as either differentiated carcinomas forming tubular or papillary structures or poorly differentiated carcinomas including mucinous adenocarcinoma, in which such structures are inconspicuous. Poorly differentiated colorectal carcinomas are quite rare, comprising only 3–5% of all colorectal carcinomas. It is well known that mucinous carcinoma is frequently observed in colorectal cancer with genetic instability, but the difference in genetic pathways between these histological types is mostly unknown because of the very small number of cases (Risio *et al*, 1996).

In this study, we investigated the methylation status of *CDH13* in colorectal cancers and found that almost all (83%) poorly differentiated colorectal cancers presented *CDH13* methylation, while only 28% of other differentiated colorectal cancers did. *CDH13,* as a member of the cadherin family, would be a cell surface glycoprotein responsible for cell adhesion. Therefore, it is conceivable that *CDH13* is inactivated in colorectal cancers by promoter methylation, leading to cancer cell dissociation, which is a characteristic of poorly differentiated carcinoma. Recently, it was reported that most poorly differentiated colorectal carcinomas no longer express E-cadherin, another cadherin family member, because of promoter methylation (Kanazawa *et al*, 2002). Moreover, another study reported that the E-cadherin promoter frequently underwent hypermethylation in human gastric cancers, particularly those of the undifferentiated histologic subtype (Tamura *et al*, 2000). These results support the notion that the inactivation of cadherin family genes would be a critical event in the scattering of carcinoma cells scattered because they code for proteins responsible for selective cell recognition and adhesion.

As described, the methylation of *CDH13* gene would not be complete, suggesting that the *CDH13* gene expression has not been inhibited completely in primary colorectal cancers. Zheng *et al* (2001) reported previously that the partial methylation pattern was associated with relatively low levels of *p14ARF* in colorectal cancer cell lines. *p14ARF* mRNA was expressed at extremely low levels in fully methylated cell lines. *p14ARF* expression in the partial methylated LoVo cell line was intermediate. Moreover, partial methylation of *p14ARF* was the most common pattern observed in primary colorectal cancers. Taken together, it was suggested that the level of *CDH13* gene expression might be also controlled by methylation in colorectal cancers.

Recent studies have shown that it is possible to reverse epigenetic changes and restore gene function to a cell. Treatment with DNA methylation inhibitors can restore the activities of the *CDH13* gene and decrease the growth rate of cancer cells. The administration of drugs such as cytosine analogues might soon enable the functional restoration of these tumour suppressor genes and slow the rate of colorectal cancer progression.
